# Dietary Salt Restriction and Adherence to the Mediterranean Diet: A Single Way to Reduce Cardiovascular Risk?

**DOI:** 10.3390/jcm13020486

**Published:** 2024-01-16

**Authors:** Lanfranco D’Elia, Pasquale Strazzullo

**Affiliations:** Department of Clinical Medicine and Surgery, “Federico II” University of Naples Medical School, 80131 Naples, Italy; lanfranco.delia@unina.it

**Keywords:** salt, sodium, Mediterranean dietary pattern, cardiovascular risk, blood pressure

## Abstract

The dietary restriction of salt intake and the adhesion to Mediterranean dietary patterns are among the most recommended lifestyle modifications for the prevention of cardiovascular diseases. A large amount of evidence supports these recommendations; indeed, several studies show that a higher adherence to Mediterranean dietary patterns is associated with a reduced risk of cardiovascular disease. Likewise, findings from observational and clinical studies suggest a causal role of excess salt intake in blood pressure increase, cardiovascular organ damage, and the incidence of cardiovascular diseases. In this context, it is also conceivable that the beneficial effects of these two dietary patterns overlap because Mediterranean dietary patterns are typically characterized by a large consumption of plant-based foods with low sodium content. However, there is little data on this issue, and heterogeneous results are available on the relationship between adherence to salt restriction and to Mediterranean dietary patterns. Thus, this short review focuses on the epidemiological and clinical evidence of the relationship between the adherence to Mediterranean dietary patterns and dietary salt restriction in the context of cardiovascular risk.

## 1. Introduction

Non-communicable diseases (NCDs) and, in particular, cardiovascular diseases (CVD) are the leading causes of death globally [1], and their reduction is a health priority [2]. In turn, high blood pressure (BP) and unhealthy diets are major causes of CVD [1,2].

In this context, the role of dietary salt (i.e., sodium chloride) has been extensively studied in relation to its effects on CVD. In particular, a large body of evidence supports a causal role of excess salt intake in the increase in BP with age, in the development of hypertension [3], and, eventually, in the incidence of CVD [3,4]. Epidemiological evidence regarding the strong relationship between salt intake, BP, and hypertension was provided over 30 years ago [5], and was confirmed thereafter by numerous studies [6,7]. In particular, recent results of the CARDIA study indicated that in a sample of initially normotensive young participants (mean age: 30 years), an average salt consumption of 14 g per day was associated with a 53% higher risk of hypertension than with a consumption of approximately 3 g per day over a 25-year follow-up period [8].

Excess salt intake has detrimental effects on endothelial function, contributes to the salt sensitivity of BP, activates the sympathetic nervous system, and is involved in the inflammatory response, modulating innate and adaptive immunity [9,10]. There is experimental evidence of structural and functional alterations induced by high-salt regimens in the arterial wall above and beyond the effect of high BP [10]. 

The results of intervention studies are in agreement with observational and experimental data. For instance, robust evidence regarding the effect of changes of dietary salt intake on BP was provided by a seminal study, which showed a significant increment of BP after switching a group of chimpanzees from their habitual low-salt diet to a high-salt diet for six months, and then a switch of BP back to normal when they returned to their usual low-salt regimen [11]. Thereafter, several controlled clinical trials examining the effect of dietary salt intake on BP have been conducted in humans, and their results have been the object of meta-analyses showing a favorable effect of salt intake reduction on BP in different settings (e.g., participants with and without hypertension, diabetic patients, and patients with renal disorders) [12,13,14,15,16,17]. 

In addition to the effects on BP, many studies have indicated that elevated salt intake may promote target organ damage and, conversely, several clinical trials have shown that salt restriction leads to an improvement in arterial stiffness [18], urinary albumin excretion [19], central blood pressure [18], and left ventricular mass [20,21,22]. 

Given the well-proven relationship between high BP and coronary, cerebrovascular, and renal outcomes, it is expected that salt intake in turn affects the incidence of cardiovascular disorders. Indeed, many longitudinal studies have detected a direct association between salt intake and CVD, and in particular with stroke risk [3,23,24]. 

Recently, the Mediterranean diet has been recognized as one of the dietary models more in keeping with the model of planetary diet conceived by the EAT–Lancet Commission as a diet for the Anthropocene [25]. The potential beneficial effects of a high level of adhesion to the Mediterranean dietary pattern (MDP) on CVD were hypothesized as early as in the 1950s [26]. That observation has inspired countless studies in which a higher degree of adhesion to the MDP was associated with a reduced risk of all-cause mortality and CVD [27,28]. In this regard, a recent meta-analysis of the effects of seven popular structured dietary patterns has shown that only the MDP substantially reduces all-cause mortality, non-fatal myocardial infarction, and stroke rates [28]. The benefit toward the risk of stroke was also reported by a previous meta-analysis showing that a higher adhesion to the MDP was associated with a lower risk of stroke in both Mediterranean and non-Mediterranean populations, and for both the ischemic and hemorrhagic types of stroke [29]. Likewise, yet another meta-analysis of 16 prospective cohort studies including only women detected a significant association between a higher adherence to the MDP and a lower incidence of total cardiovascular and coronary events, as well as total mortality, and a weaker association between MDP adhesion and the risk of stroke [30]. A very recent observational study, including approximately 2000 middle-aged male and female Greek participants, showed that those who sustained a high degree of adhesion to the MDP over the years had the lowest 20-year CVD risk [31]. In agreement with these results, other prospective cohort studies provided evidence that high adherence to the MDP improves survival in people with a history of CVD [32]. In keeping with the evidence on the relationship between MDP and the risk of cardiovascular events, several studies have also provided evidence of the association between the MDP and numerous cardiovascular risk factors. Thus, adhesion to the MDP was associated with beneficial changes in body weight, waist circumference, BP, insulin resistance, lipid profile, and flow-mediated arteriolar dilation [33]. The favorable effect of the MDP on BP was also found by two meta-analyses that detected a lower average systolic BP in participants with a higher degree of adhesion to the MDP compared to the lower-adhesion group [34,35]. In line with these results are those of another meta-analysis including 58 studies with finding of significantly lower values of waist circumference and serum triglyceride, and higher values of HDL-cholesterol in the high-adhesion MDP individuals [36]. Finally, based on 16 prospective studies, a systematic review found that the greatest adhesion to the MDP was significantly associated with a reduced risk of type 2 diabetes through a non-linear relationship [37].

Given that MDP is characterized by a large consumption of fruits, vegetables, whole grains, legumes, fish, monounsaturated fats (in particular olive oil), and nuts [38], there are many possible biological pathways whereby a variety of substances in the MDP could exert a beneficial role on CVD; for instance, dietary fiber, vitamins, minerals, and polyphenols content may benefit an altered metabolism of glucose and lipids as well as abdominal adiposity, high BP, and cardiovascular organ damage [39]. Based on these considerations, it is conceivable that a lower sodium intake might play a contributory role in the beneficial effects of the MDP, since the sodium content of natural plant-based foods is typically low. Nevertheless, few data are available on this issue and heterogeneous results on the relationship between adherence to salt intake and adherence to the MDP are available [40,41,42,43,44,45]. Therefore, we carried out a short narrative review focusing on the epidemiological and clinical evidence of the relationship between adhesion to the MDP and dietary salt intake in relation to CVD risk.

## 2. Epidemiological Evidence

The first strong epidemiological evidence of the direct association between salt intake and BP was provided by the results of the INTERSALT study, showing that the higher the habitual salt intake, the higher the average BP increase with age and the prevalence of hypertension in different populations around the world [5]. The detrimental effect of salt intake on cardiovascular risk was documented by several meta-analyses, in which an unequivocal association was detected with CVD, and in particular with stroke risk. The first of such meta-analyses, including prospective studies of samples of general populations, indicated a direct and significant association between higher salt intake and incidence of CVD [4]. The analysis, including 170,000 participants and more than 11,000 vascular events, showed a 23% greater risk of stroke and a 17% greater risk of total cardiovascular events for an average difference in salt intake of 5 g of salt per day. Further meta-analyses substantially confirmed these trends, despite some differences in the number of studies included in the analyses [3,23]. 

By the same token, looking at the epidemiological evidence on MDP, a number of prospective studies were carried out on the association between adhesion to the MDP and health outcomes. A recent systematic review has suggested that a high degree of adhesion to the MDP was associated with lower mortality rates in samples of general populations and in patients with previous CVD [27]. In addition, a separate evaluation of six prospective studies indicated that, following MDP, the risk of CVD (i.e., coronary artery disease, stroke, and cardiovascular mortality) decreased; in particular, the reduction in risk ranged from a hazard ratio of 0.44 to 0.71. The association was found when the adhesion to the MDP was expressed both as a continuous and as a dichotomous variable.

Despite this consolidated individual role of low salt intake and MDP adherence in CVD, and the potential synergistic effect of the combination of the two dietary measures, no epidemiological investigation explored this issue. Nevertheless, a few studies tried to assess the relationship between adhesion to the MDP and of the salt content of the diet in this context [40,41,42,43,44,45] (Table 1). 

In a large Spanish population (n = 17,197), a comparison of the mineral content of the MDP and Western dietary patterns was made by a validated semi-quantitative questionnaire with 136 food items [40]. After stratification by quintiles of adhesion to the MDP or to Western dietary patterns, the highest quintile of MDP adhesion was associated with the lowest salt intake after adjustment for total energy intake, with a progressive inverse trend from the first to the last quintile. By contrast, the highest quintile of adhesion to a Western dietary pattern was associated with the highest salt intake, with a progressive direct trend from the first to the last quintile.

As a confirmation that adhesion to the MDP is compatible with a relatively low salt intake, there are the findings of a validated online self-administered questionnaire on an opportunistic large Italian population (n = 11,618), which explored the salt and health-related knowledge and behavior as well as the degree of adhesion to the MDP [42]. The survey indicated that both the degree of knowledge and the behavior regarding salt intake were significantly and positively correlated with the level of MDP adhesion, which suggests that nutritional information and good eating habits tend to go hand in hand. Of note, although the sample showed a decent level of knowledge and behavior regarding salt, remarkable differences in the degree of knowledge were detected in relation to socio-demographic markers, with a low level among adolescents, less educated people, and people with a low level of employment or that were unemployed.

On the other hand, other surveys were not in keeping with the above findings. Thus, the results of a study including 719 adults from a Northern Italy community and based on EPIC-FFQ showed that MDP adhesion had little influence on salt intake [43]. However, the assessment of salt intake in this study may have been biased because discretionary salt use was not considered.

A similar trend was also found in surveys that assessed salt intake via 24 h urinary excretion (the gold standard of the salt intake assessment). The results of a cross-sectional survey on a small sample of healthy Greek participants (n = 252) indicated no significant relationship between salt intake and MDP adherence (assessed using an 11-item questionnaire) [41]. Likewise, the results of a study on a small sample of middle-aged Portuguese volunteers (n = 102) showed no association between 24 h urinary sodium excretion and MDP adhesion, even after adjustment for confounding variables. In particular, a high degree of adhesion to the MDP was not associated with a low level of salt intake [45]. In addition, a survey on a sample of older Portuguese participants (n = 1321, ≥65 years) indicated that an excessive salt intake (assessed by 24 h urine excretion) was significantly associated with high adherence to the MDP in men, although with extremely wide confidence intervals (OR = 1.94; 95% CI: 1.03–3.65), while no such association was detected in women (OR = 0.91; 95% CI: 0.62–1.34) [44]. 

## 3. Clinical Evidence

Several intervention studies detected a direct effect of salt intake on cardiovascular risk through BP-mediated and BP-independent effects. Among the most robust trials, the TOHP I [46] and TOHP II [47], including patients with pre-hypertension at baseline, showed a significant decrease in BP in the group with salt restriction when compared to the control group. In addition, in the same studies, after 10–15 years of follow-up, a significantly lower risk of CVD was observed in the participants randomized to the salt-reduction group compared to controls [48]. This association was confirmed at the next follow-up, in which a significant linear association between salt intake and mortality was detected [49]. These favorable results of dietary salt restriction on BP were confirmed in several meta-analyses [12,14,17]. In particular, a linear dose–response relationship between the salt intake and the magnitude of the decrease in BP was also detected, with a greater effect in patients with hypertension compared to individuals without hypertension [12]. Intervention studies also support the detrimental effect of excess salt intake on target organ damage, even independently of increases in BP. For instance, the main results of a meta-analysis including 11 randomized controlled trials (RCT) and 431 participants detected a statistically significant effect of reduction in salt intake on arterial stiffness (expressed as carotid–femoral pulse wave velocity): an average weighted reduction of 5.2 g of salt intake per day led to an approximately 3% decrease in arterial stiffness, at least in part independently of the changes in BP [18]. Likewise, a meta-analysis of 23 studies including 516 participants suggested that salt restriction markedly reduces albumin excretion, a risk factor for the development and progression of renal disease [19]. In particular, an average reduction in salt intake (5.4 g of salt per day) was significantly associated with a 32% reduction in urinary albumin excretion. Furthermore, in line with this trend, the results of another meta-analysis showed the effect of salt restriction on central BP parameters (independent cardiovascular risk factors). An analysis including 14 studies and 457 participants, with an intervention time ranging between 1 and 13 weeks, detected that salt restriction led to a significant reduction in central BP parameters (e.g., the augmentation index, central systolic BP, and central pulse pressure) [16]. 

A milestone of the clinical trials examining the effects of particular dietary patterns on cardiovascular risk, which focused on the role of salt restriction, was the Dietary Approaches to Stop Hypertension (DASH) with sodium restriction trial, in which a reduction in the salt intake of the typical current American diet significantly reduced BP in hypertensive patients on top of other dietary modifications, without clinically apparent adverse effects [50]. Subsequent trials confirmed the DASH-sodium trial’s benefit and revealed a greater beneficial effect on BP than during a DASH diet without salt restriction [51,52]. 

A number of studies were carried out to explore the effect of the MDP, a “natural” dietary pattern, on cardiovascular risk. An interesting and pioneering intervention study was carried out in the Cilento region (a representative place of the MDP in southern Italy), in which 57 non-hospitalized normotensive volunteers underwent a six-week isocaloric dietary intervention with a 70% increase in energy from saturated fatty acids and a corresponding decrease in carbohydrate and mono-unsaturated fat [53]. At the end of this intervention period, BP was significantly increased. After returning to their customary diet (i.e., MDP characterized by olive oil as the main source of fat, high vegetable and fruit consumption, and low animal protein and carbohydrate intake), BP returned to baseline, without changes in body weight throughout the study. 

The PREvención con DIeta MEDiterránea (PREDIMED) study, a well-known intervention trial of the MDP including nearly 7500 participants with high cardiovascular risk, evaluated the effects of two MDPs (one with extra-virgin olive oil supplementation and the other with mixed-nuts supplementation) in comparison with a control low-fat diet, without any caloric or salt restriction, on the main CV outcomes [54]. After a median follow-up period of 3.8 years, greater reductions in BP were found for both MDPs compared with the control diet. However, only diastolic BP remained significant after multivariate adjustment.

Recently, a meta-analysis including 35 studies on the effect of the MDP on BP confirmed the beneficial effect of the MDP on BP. In this analysis, the MDP, compared to the usual diet and all other active intervention diets, significantly reduced systolic BP (−1.5 mmHg) and diastolic BP (−0.9 mmHg), in participants with or without hypertension, regardless of baseline BP levels [35]. However, this effect was only confirmed when the MDP was compared to the usual diet, while when compared to all other active intervention diets, the effect was lost.

Although a large number of studies explored the effect of the MDP or salt intake on cardiovascular risk, few intervention studies assessed the effect of salt intake in the context of the MDP and vice versa. 

The results of a supplemental analysis of the PREDIMED study supported the add-on beneficial role of salt restriction in the context of the MDP; a decrease in sodium intake to 2300 mg per day was associated with reduced total mortality; by contrast, a positive association was found between an increase in sodium intake to 2300 mg per day and total mortality in a population at high risk of CVD [55]. Hence, in consideration of the study design, reducing sodium intake to 2300 mg/day seems associated with an enhanced beneficial effect of the MDP on CVD in comparison with those who did not reduce sodium intake.

Noteworthily, a recent RCT evaluated the BP effects of a three-month dietary intervention implementing salt restriction either alone or in the context of the MDP and the DASH diet, in adults with high normal BP or grade 1 hypertension [56]. A total of 204 Greek participants were included in the final analysis of the trial, stratified into a control group, a salt-restriction group, a MDP with salt restriction group, and a DASH diet with salt restriction group. The main results of this RCT indicated a greater and significant reduction in the office systolic BP in the MDP with salt restriction group compared to all other study groups (vs. control group: −15.1 mmHg; vs. salt restriction group: −7.5 mmHg; vs. DASH diet with salt restriction group: −3.2 mmHg). However, the MDP with salt restriction and DASH diet with salt restriction groups did not differ concerning the office diastolic BP. In addition, both MDP and DASH diets were more efficient in the reduction of BP than salt restriction alone. 

## 4. Discussion 

### 4.1. Dietary Salt Intake, BP Salt-Sensitivity and Cardiovascular Outcomes

The bulk of evidence provided by experimental, clinical and epidemiological studies supports the concept that excess salt intake has detrimental effects on BP and may promote organ damage mainly due to the rise in BP but also via additional BP-independent mechanisms. Although the majority of subjects experience a decrease in BP upon reduction of salt intake, in particular those with higher BP, there is a substantial inter-individual difference in the degree of the BP response to similar changes in salt intake [57], a phenomenon referred to as BP salt-sensitivity. A number of factors affect BP salt-sensitivity: among these the renin–angiotensin–aldosterone system (RAAS) response to changes in salt intake plays a major role in this regard [58]. In general, at high salt consumption, RAAS activity is suppressed, and this attenuates the tendency towards an increase in BP consequent to the increase in the extracellular fluid volume. However, in some relatively frequent conditions (e.g., obesity and type 2 diabetes), RAAS suppression may be blunted, leading to an increased BP salt-sensitivity. Moreover, activation of the sympathetic nervous system (SNS) may also contribute to the BP salt-sensitivity [59,60]. 

Many experimental studies showed that high salt consumption may affect arterial structure and function also partly independently of an increase in BP. Indeed, it has been suggested that high salt intake may have unfavorable effects on endothelial activity by increasing the production of transforming growth factor-beta 1 (TGF-b1) [61,62,63], reducing the bioavailability of nitric oxide (NO) [63], and decreasing the expression of endothelial NO synthase (eNOS) [64]. It has been shown that high endothelial sodium concentration impairs NO production and reduces its cellular plasticity [65]. Likewise, sodium overload may decrease the endothelial glycocalyx sodium barrier and concomitantly increase the endothelial stiffness [65]. The local RAAS may be involved in these events, as high salt intake was associated with increased angiotensin II type 1 receptor (AT1-R) expression in the cardiovascular system [66] and with an increase in AT1-R density in the renal cortex [66,67]. Accordingly, the administration of an AT1-R blocker during salt loading inhibits some detrimental effects mediated by this receptor (e.g., by improving the cardiovascular function and reducing aortic collagen accumulation) [66,68]. Also, chronic inflammation during excess salt intake may contribute to cardiovascular damage [9,69]. Indeed, mechanical stimuli due to high BP lead to a vascular inflammatory response, which involves both innate and adaptive immunity [9,70]. 

### 4.2. Benefits, Cost-Effectiveness, and Safety of Moderate Dietary Salt Reduction

Unfortunately, notwithstanding the educational campaigns in favor of salt intake reduction to values below 5 g per day (2 g per day of sodium) based on WHO recommendations, in most countries worldwide the habitual average salt intake largely exceeds this level [71]. To support the benefits of dietary salt reduction, numerous cost-effectiveness analyses indicated that a reduction of dietary salt at the population level is highly cost-effective and cost-saving in reducing CVD [72,73,74]. Indeed, an analysis of 183 countries found that a government “soft regulation” policy intervention to reduce national sodium consumption by 10% over 10 years was projected to be highly cost-effective (<1 × gross domestic product (GDP) per capita per disability life year (DALY) saved) in most countries [74]. Therefore, hundreds of thousands of deaths, and millions of DALYs, could be avoided each year, at a low cost. 

Side effects upon reduction of salt intake to the levels recommended by the WHO are extremely rare in healthy individuals, since homeostatic mechanisms are very effective in maintaining the plasma sodium concentration within a narrow range [75]. On the other hand, more severe chronic restriction and/or the presence of disorders affecting water and electrolyte homeostasis may lead to symptomatic hyponatremia (i.e., a blood sodium level lower than 135 mmol/L), as reported in patients with severe vomiting and/or diarrhea, heart failure, excessive intake of diuretics, kidney disease, polydipsia, and liver cirrhosis [75]. 

### 4.3. Association of Low Salt Intake and Adhesion to the MDP

In addition to dietary salt intake reduction, also the benefits of a high degree of adhesion to the MDP are undoubtable. Indeed, a moderate reduction of salt intake and high adhesion to the MDP are both highly recommended lifestyle modifications for the prevention of CVD. In particular, the most recent European Society of Hypertension guidelines indicate both salt reduction and improvement of MDP adhesion as important measures for the prevention and management of hypertension [76]. In addition, the results of a recent trial support a possible synergic effect of these two interventions, inasmuch that MDP with salt restriction provided a greater reduction in systolic BP than either intervention alone in patients with high-normal or grade 1 hypertension [56]. 

In the natural foods that are prevalent in plant-based diets, such as the MDP, the salt content is low or very low [77]. In spite of this, however, the epidemiological evidence in favor of a direct association between the degree of adhesion to the Mediterranean diet and low habitual salt intake is not unequivocal: a larger use of discretionary salt in cooking and/or at the table and the higher salt content of commercially available processed foods provide a reasonable explanation for these heterogenous results. A recent Italian study supports this explanation, indicating that cereal-based products, including bread, represent a major source of daily non-discretionary salt intake [78]. 

In a way similar to the case of salt intake reduction, and in spite of the universal recognition of the benefits of plant-based diets such as the Mediterranean diet, the degree of adhesion to this model is still low, as documented by a recent survey of the CREA—Research Centre for Food and Nutrition in Rome, Italy [79]. Indeed, ecological studies in countries of the Mediterranean area indicated a shift in dietary patterns from the 1960s to the 2000s, especially in terms of an increase in animal protein and fat intake and a reduction in fresh plant-based foods [80,81]. This scenario is also supported by the cost of healthy foods (e.g., fresh fruit and vegetables), which may be definitely more expensive compared with processed and ultra-processed foods typical of Western dietary patterns [82]; indeed, a greater prevalence of a lower socioeconomic conditions is associated with a more energy-dense low-quality diet [83]. These unfavorable dietary changes, in turn, contribute to an increase in the incidence of excess body weight and other risk factors for non-communicable chronic diseases, and in particular cardiometabolic disorders, starting from childhood [84,85].

### 4.4. Conclusions, Limitations of the Study, and Future Perspectives

This short review summarized the benefits of dietary salt reduction and high adhesion to the MDP for cardiometabolic health, and tried to highlight their potential synergistic effects. Nevertheless, it has a few potential limitations, the first of which is the limited data available on the combined effect of these two major dietary preventive measures on CVD risk. We were able to retrieve only few and non-univocal data on the association between the adherence to the MDP and to reduced salt intake. Indeed, whereas a few epidemiological studies reported the expected inverse association between adhesion to the MDP and dietary salt intake [40,42], other studies did not detect any association. Conceivably, the different features of the studies available on this subject, among which the different questionnaires used to evaluate adhesion to the MDP and the different methods adopted for the assessment of habitual salt intake, may have contributed to these heterogeneous results. Therefore, definite conclusions cannot be drawn both for the strength of the combined effect of the two dietary measures on CVD risk and with regard to the association between adhesion to the MDP and salt intake reduction. There is clearly a need to adopt more effective strategies aiming to promote the increase of healthy food availability and of healthy eating patterns, which are key elements of preventive medicine, addressing in particular young people, in order to reduce the persistently high burden of CVD.

## Figures and Tables

**Table 1:** Characteristics of the studies reporting the relationship between Mediterranean dietary patterns and salt intake in adult general populations.

First Author (Year) [Ref]	Country	Participants (n)	MDP Adhesion Assessment Method	Salt Intake Assessment	Results
Serra-Majem et al. (2009) [40]	Europe (Spain)	17,197	Semi-quantitative FFQ (136 food items)	Semi-quantitative FFQ (136 food items)	The highest quintile of adhesion to the MDP was associated with the lowest salt intake (adjusted for total energy intake)
Vasara et al. (2017) [41]	Europe (Greece)	252	FFQ (11 food items)	24 h urine collection	No association between degree of adhesion to the MDP and salt intake
Iaccarino Idelson et al. (2020) [42]	Europe (Italy)	11,618	Self-administered questionnaire (4 food items)	Self-administered questionnaire (31 food items)	The level of MDP adhesion was significantly and positively correlated with the degree of knowledge and behavior about salt intake
Malavolti et al. (2021) [43]	Europe (Italy)	719	Semi-quantitative FFQ (EPIC—188 food items)	Semi-quantitative FFQ (EPIC—188 food items)	No association between adhesion to the MDP and salt intake
Moreira et al. (2021) [44]	Europe (Portugal)	1321	Semi-quantitative FFQ (14 food items)	24 h urine collection	In multivariate analysis, salt intake was positively associated with adhesion to the MDP in men. No such association was found in women
Viroli et al. (2021) [45]	Europe (Portugal)	102	Semi-quantitative FFQ (82 food items)	24 h urine collection	No association between adhesion to the MDP and salt intake

FFQ: food frequency questionnaire; MDP: Mediterranean dietary patterns.

## References

[1] GBD 2019 Risk Factors Collaborators (2020). Global burden of 87 risk factors in 204 countries and territories, 1990–2019: A systematic analysis for the Global Burden of Disease Study 2019. Lancet.

[2] GBD 2017 Diet Collaborators (2019). Health effects of dietary risks in 195 countries, 1990–2017: A systematic analysis for the Global Burden of Disease Study 2017. Lancet.

[3] Aburto N.J., Ziolkovska A., Hooper L., Elliott P., Cappuccio F.P., Meerpohl J.J. (2013). Effect of lower sodium intake on health: Systematic review and meta-analyses. BMJ.

[4] Strazzullo P., D’Elia L., Kandala N.B., Cappuccio F.P. (2009). Salt intake, stroke, and cardiovascular disease: Meta-analysis of prospective studies. BMJ.

[5] Intersalt Cooperative Research Group (1988). Intersalt: An international study of electrolyte excretion and blood pressure. Results for 24–hour urinary sodium and potassium excretion. BMJ.

[6] Zhou B., Stamler J., Dennis B., Moag-Stahlberg A., Okuda N., Robertson C., Zhao L., Chan Q., Elliott P., for the INTERMAP Research Group (2003). Nutrient intakes of middle-aged men and women in China, Japan, United Kingdom, and United States in the late 1990s: The INTERMAP study. J. Hum. Hypertens..

[7] Khaw K.T., Bingham S., Welch A., Luben R., O’Brien E., Wareham N., Day N. (2004). Blood pressure and urinary sodium in men and women: The Norfolk Cohort of the European Prospective Investigation into Cancer (EPIC-Norfolk). Am. J. Clin. Nutr..

[8] Hisamatsu T., Lloyd-Jones D.M., Colangelo L.A., Liu K. (2021). Urinary sodium and potassium excretions in young adulthood and blood pressure by middle age: The Coronary Artery Risk Development in Young Adults (CARDIA) Study. J. Hypertens..

[9] Miyauchi H., Geisberger S., Luft F.C., Wilck N., Stegbauer J., Wiig H., Dechend R., Jantsch J., Kleinewietfeld M., Kempa S. (2023). Sodium as an Important Regulator of Immunometabolism. Hypertension.

[10] D’Elia L., Strazzullo P. (2022). Isolated systolic hypertension of the young and sodium intake. Minerva Med..

[11] Denton D., Weisinger R., Mundy N.I., Wickings E.J., Dixson A., Moisson P., Pingard A.M., Shade R., Carey D., Ardaillou R. (1995). The effect of increased salt intake on blood pressure of chimpanzees. Nat. Med..

[12] Filippini T., Malavolti M., Whelton P.K., Naska A., Orsini N., Vinceti M. (2021). Blood Pressure Effects of Sodium Reduction: Dose-Response Meta-Analysis of Experimental Studies. Circulation.

[13] Ren J., Qin L., Li X., Zhao R., Wu Z., Ma Y. (2021). Effect of dietary sodium restriction on blood pressure in type 2 diabetes: A meta-analysis of randomized controlled trials. Nutr. Metab. Cardiovasc. Dis..

[14] Huang L., Trieu K., Yoshimura S., Neal B., Woodward M., Campbell N.R.C., Li Q., Lackland D.T., Leung A.A., Anderson C.A.M. (2020). Effect of dose and duration of reduction in dietary sodium on blood pressure levels: Systematic review and meta-analysis of randomised trials. BMJ.

[15] Cole N.I., Swift P.A., He F.J., MacGregor G.A., Suckling R.J. (2019). The effect of dietary salt on blood pressure in individuals receiving chronic dialysis: A systematic review and meta-analysis of randomised controlled trials. J. Hum. Hypertens..

[16] D’Elia L., La Fata E., Giaquinto A., Strazzullo P., Galletti F. (2020). Effect of dietary salt restriction on central blood pressure: A systematic review and meta-analysis of the intervention studies. J. Clin. Hypertens..

[17] He F.J., Li J., Macgregor G.A. (2013). Effect of longer-term modest salt reduction on blood pressure: Cochrane systematic review and meta-analysis of randomized trials. BMJ.

[18] D’Elia L., Galletti F., La Fata E., Sabino P., Strazzullo P. (2018). Effect of dietary sodium restriction on arterial stiffness: Systematic review and meta-analysis of the randomized controlled trials. J. Hypertens..

[19] D’Elia L., Rossi G., Schiano di Cola M., Savino I., Galletti F., Strazzullo P. (2015). Meta-Analysis of the Effect of Dietary Sodium Restriction with or without Concomitant Renin-Angiotensin-Aldosterone System-Inhibiting Treatment on Albuminuria. Clin. J. Am. Soc. Nephrol..

[20] Kupari M., Koskinen P., Virolainen J. (1994). Correlates of left ventricular mass in a population sample aged 36 to 37 years. Focus on lifestyle and salt intake. Circulation.

[21] Schmieder R.E., Messerli F.H., Garavaglia G.E., Nunez B.D. (1988). Dietary salt intake. A determinant of cardiac involvement in essential hypertension. Circulation.

[22] Jula A.M., Karanko H.M. (1994). Effects on left ventricular hypertrophy of long-term non-pharmacological treatment with sodium restriction in mild-to-moderate essential hypertension. Circulation.

[23] Iacoviello L., Bonaccio M., Cairella G., Catani M.V., Costanzo S., D’Elia L., Giacco R., Rendina D., Sabino P., Savini I. (2018). Diet and primary prevention of stroke: Systematic review and dietary recommendations by the ad hoc Working Group of the Italian Society of Human Nutrition. Nutr. Metab. Cardiovasc. Dis..

[24] Jayedi A., Ghomashi F., Zargar M.S., Shab-Bidar S. (2019). Dietary sodium, sodium-to-potassium ratio, and risk of stroke: A systematic review and nonlinear dose-response meta-analysis. Clin. Nutr..

[25] Willett W., Rockström J., Loken B., Springmann M., Lang T., Vermeulen S., Garnett T., Tilman D., DeClerck F., Wood A. (2019). Food in the Anthropocene: The EAT-Lancet Commission on healthy diets from sustainable food systems. Lancet.

[26] Keys A. (1995). Mediterranean diet and public health: Personal reflections. Am. J. Clin. Nutr..

[27] Laffond A., Rivera-Picón C., Rodríguez-Muñoz P.M., Juárez-Vela R., Ruiz de Viñaspre-Hernández R., Navas-Echazarreta N., Sánchez-González J.L. (2023). Mediterranean Diet for Primary and Secondary Prevention of Cardiovascular Disease and Mortality: An Updated Systematic Review. Nutrients.

[28] Karam G., Agarwal A., Sadeghirad B., Jalink M., Hitchcock C.L., Ge L., Kiflen R., Ahmed W., Zea A.M., Milenkovic J. (2023). Comparison of seven popular structured dietary programmes and risk of mortality and major cardiovascular events in patients at increased cardiovascular risk: Systematic review and network meta-analysis. BMJ.

[29] Chen G.C., Neelakantan N., Martín-Calvo N., Koh W.P., Yuan J.M., Bonaccio M., Iacoviello L., Martínez-González M.A., Qin L.Q., van Dam R.M. (2019). Adherence to the Mediterranean diet and risk of stroke and stroke subtypes. Eur. J. Epidemiol..

[30] Pant A., Gribbin S., McIntyre D., Trivedi R., Marschner S., Laranjo L., Mamas M.A., Flood V., Chow C.K., Zaman S. (2023). Primary prevention of cardiovascular disease in women with a Med-iterranean diet: Systematic review and meta-analysis. Heart.

[31] Georgoulis M., Damigou E., Chrysohoou C., Barkas F., Anastasiou G., Kravvariti E., Tsioufis C., Liberopoulos E., Sfikakis P.P., Pitsavos C. (2024). Mediterranean diet trajectories and 20-year incidence of cardiovascular disease: The ATTICA cohort study (2002-2022). Nutr. Metab. Cardiovasc. Dis..

[32] Tang C., Wang X., Qin L.Q., Dong J.Y. (2021). Mediterranean Diet and Mortality in People with Cardiovascular Disease: A Meta-Analysis of Prospective Cohort Studies. Nutrients.

[33] Papadaki A., Nolen-Doerr E., Mantzoros C.S. (2020). The Effect of the Mediterranean Diet on Metabolic Health: A Systematic Review and Meta-Analysis of Controlled Trials in Adults. Nutrients.

[34] Bakaloudi D.R., Chrysoula L., Leonida I., Kotzakioulafi E., Theodoridis X., Chourdakis M. (2021). Impact of the level of adherence to the Mediterranean Diet on blood pressure: A systematic review and meta-analysis of observational studies. Clin. Nutr..

[35] Filippou C.D., Thomopoulos C.G., Kouremeti M.M., Sotiropoulou L.I., Nihoyannopoulos P.I., Tousoulis D.M., Tsioufis C.P. (2021). Mediterranean diet and blood pressure reduction in adults with and without hypertension: A systematic review and meta-analysis of randomized controlled trials. Clin. Nutr..

[36] Bakaloudi D.R., Chrysoula L., Kotzakioulafi E., Theodoridis X., Chourdakis M. (2021). Impact of the Level of Adherence to Mediterranean Diet on the Parameters of Metabolic Syndrome: A Systematic Review and Meta-Analysis of Observational Studies. Nutrients.

[37] Sarsangi P., Salehi-Abargouei A., Ebrahimpour-Koujan S., Esmaillzadeh A. (2022). Association between Adherence to the Mediterranean Diet and Risk of Type 2 Diabetes: An Updated Systematic Review and Dose-Response Meta-Analysis of Prospective Cohort Studies. Adv. Nutr..

[38] Widmer R.J., Flammer A.J., Lerman L.O., Lerman A. (2015). The Mediterranean Diet, its Components, and Cardiovascular Disease. Am. J. Med..

[39] Farias-Pereira R., Zuk J.B., Khavaran H. (2023). Plant bioactive compounds from Mediterranean diet improve risk factors for metabolic syndrome. Int. J. Food Sci. Nutr..

[40] Serra-Majem L., Bes-Rastrollo M., Román-Viñas B., Pfrimer K., Sánchez-Villegas A., Martínez-González M.A. (2009). Dietary patterns and nutritional adequacy in a Mediterranean country. Br. J. Nutr..

[41] Vasara E., Marakis G., Breda J., Skepastianos P., Hassapidou M., Kafatos A., Rodopaios N., Koulouri A.A., Cappuccio F.P. (2017). Sodium and Potassium Intake in Healthy Adults in Thessaloniki Greater Metropolitan Area-The Salt Intake in Northern Greece (SING) Study. Nutrients.

[42] Iaccarino Idelson P., D’Elia L., Cairella G., Sabino P., Scalfi L., Fabbri A., Galletti F., Garbagnati F., Lionetti L., Paolella G. (2020). Salt and Health: Survey on Knowledge and Salt Intake Related Behaviour in Italy. Nutrients..

[43] Malavolti M., Naska A., Fairweather-Tait S.J., Malagoli C., Vescovi L., Marchesi C., Vinceti M., Filippini T. (2021). Sodium and Potassium Content of Foods Consumed in an Italian Population and the Impact of Adherence to a Mediterranean Diet on Their Intake. Nutrients.

[44] Moreira S., Moreira P., Sousa A.S., Guerra R.S., Afonso C., Santos A., Borges N., Amaral T.F., Padrão P. (2021). Urinary Sodium Excretion and Adherence to the Mediterranean Diet in Older Adults. Nutrients.

[45] Viroli G., Gonçalves C., Pinho O., Silva-Santos T., Padrão P., Moreira P. (2021). High Adherence to Mediterranean Diet Is Not Associated with an Improved Sodium and Potassium Intake. Nutrients.

[46] TOHP I (1992). The effects of non-pharmacologic interventions on blood pressure of persons with high normal levels. Results of the Trials of Hypertension Prevention, Phase I. JAMA.

[47] TOHP II (1997). Effects of weight loss and sodium reduction intervention on blood pressure and hypertension incidence in overweight people with high-normal blood pressure. The Trials of Hypertension Prevention, phase II. Arch. Intern. Med..

[48] Cook N.R., Cutler J.A., Obarzanek E., Buring J.E., Rexrode K.M., Kumanyika S.K., Appel L.J., Whelton P.K. (2007). Long term effects of dietary sodium reduction on cardiovascular disease outcomes: Observational follow-up of the trials of hypertension prevention (TOHP). BMJ.

[49] Cook N.R., Appel L.J., Whelton P.K. (2016). Sodium Intake and All-Cause Mortality Over 20 Years in the Trials of Hypertension Prevention. J. Am. Coll. Cardiol..

[50] Svetkey L.P., Sacks F.M., Obarzanek E., Vollmer W.M., Appel L.J., Lin P.H., Karanja N.M., Harsha D.W., Bray G.A., Aickin M. (1999). The DASH Diet, Sodium Intake and Blood Pressure Trial (DASH-sodium): Rationale and design. DASH-Sodium Collaborative Research Group. J. Am. Diet Assoc..

[51] Appel L.J., Moore T.J., Obarzanek E., Vollmer W.M., Svetkey L.P., Sacks F.M., Bray G.A., Vogt T.M., Cutler J.A., Windhauser M.M. (1997). A clinical trial of the effects of dietary patterns on blood pressure. DASH Collaborative Research Group. N. Engl. J. Med..

[52] Sacks F.M., Svetkey L.P., Vollmer W.M., Appel L.J., Bray G.A., Harsha D., Obarzanek E., Conlin P.R., Miller E.R., Simons-Morton D.G. (2001). Effects on blood pressure of reduced dietary sodium and the dietary approaches to stop hypertension (DASH) diet. DASH-sodium collaborative research group. N. Engl. J. Med..

[53] Strazzullo P., Ferro-Luzzi A., Siani A., Scaccini C., Sette S., Catasta G., Mancini M. (1986). Changing the Mediterranean diet: Effects on blood pressure. J. Hypertens..

[54] Toledo E., Hu F.B., Estruch R., Buil-Cosiales P., Corella D., Salas-Salvadó J., Covas M.I., Arós F., Gómez-Gracia E., Fiol M. (2013). Effect of the Mediterranean diet on blood pressure in the PREDIMED trial: Results from a randomized controlled trial. BMC Med..

[55] Merino J., Guasch-Ferré M., A Martínez-González M., Corella D., Estruch R., Fitó M., Ros E., Arós F., Bulló M., Gómez-Gracia E. (2015). Is complying with the recommendations of sodium intake beneficial for health in individuals at high cardiovascular risk? Findings from the PREDIMED study. Am. J. Clin. Nutr..

[56] Filippou C., Thomopoulos C., Konstantinidis D., Siafi E., Tatakis F., Manta E., Drogkaris S., Polyzos D., Kyriazopoulos K., Grigoriou K. (2023). DASH vs. Mediterranean diet on a salt restriction background in adults with high normal blood pressure or grade 1 hypertension: A randomized controlled trial. Clin. Nutr..

[57] Strazzullo P., Galletti F., Dessì-Fulgheri P., Ferri C., Glorioso N., Malatino L., Mantero F., Manunta P., Semplicini A., Ghiadoni L. (2000). Prediction and consistency of blood pressure salt-sensitivity as assessed by a rapid volume expansion and contraction protocol. Salt-Sensitivity Study Group of the Italian Society of Hypertension. J. Nephrol..

[58] Hall J.E. (1986). Control of sodium excretion by angiotensin II: Intrarenal mechanisms and blood pressure regulation. Am. J. Physiol..

[59] Laffer C.L., Bolterman R.J., Romero J.C., Elijovich F. (2006). Effect of salt on isoprostanes in salt-sensitive essential hypertension. Hypertension.

[60] Elijovich F., Laffer C.L., Amador E., Gavras H., Bresnaham M.R., Schiffrin E.L. (2001). Regulation of plasma endothelin by salt in salt-sensitive hypertension. Circulation.

[61] Ying W.-Z., Sanders P.W. (1999). Dietary salt increases endothelial nitric oxide synthase and TGF-b1 in rat aortic endothelium. Am. J. Physiol..

[62] Ying W.-Z., Sanders P.W. (1998). Dietary salt modulates renal production of transforming growth factor-b in rats. Am. J. Physiol..

[63] Matsuoka H., Itoh S., Kimoto M., Kohno K., Tamai O., Wada Y., Yasukawa H., Iwami G., Okuda S., Imaizumi T. (1997). Asymmetrical dimethylarginine, an endogenous nitric oxide synthase inhibitor, in experimental hypertension. Hypertension.

[64] Ni Z., Vaziri N.D. (2001). Effect of salt loading on nitric oxide synthase expression in normotensive rats. Am. J. Hypertens..

[65] Oberleithner H., Peters W., Kusche-Vihrog K., Korte S., Schillers H., Kliche K., Oberleithner K. (2011). Salt overload damages the glycocalyx sodium barrier of vascular endothelium. Pflug. Arch..

[66] Nickenig G., Strehlow K., Roeling J., Zolk O., Knorr A., Bohm M. (1998). Salt induces vascular AT1 receptor overexpression in vitro and in vivo. Hypertension.

[67] Frohlich E.D. (2007). The salt conundrum: A hypothesis. Hypertension.

[68] Matavelli L.C., Zhou X., Varagic J., Susic D., Frohlich E.D. (2007). Salt loading produces severe renal hemodynamic dysfunction inde pendent of arterial pressure in spontaneously hypertensive rats. Am. J. Physiol. Heart Circ. Physiol..

[69] Zanoli L., Briet M., Empana J.P., Cunha P.G., Mäki-Petäjä K.M., Protogerou A.D., Tedgui A., Touyz R.M., Schiffrin E.L., Spronck B. (2020). Vascular consequences of inflammation: A position statement from the ESH Working Group on Vascular Structure and Function and the ARTERY Society. J. Hypertens..

[70] Ait-Oufella H., Sage A.P., Mallat Z., Tedgui A. (2014). Adaptive (T and B cells) immunity and control by dendritic cells in atherosclerosis. Circ. Res..

[71] World Health Organization (2012). Guideline: Sodium Intake for Adults and Children.

[72] Bibbins-Domingo K., Chertow G.M., Coxson P.G., Moran A., Lightwood J.M., Pletcher M.J., Goldman L. (2010). Projected effect of dietary salt reductions on future cardiovascular disease. N. Engl. J. Med..

[73] Smith-Spangler C.M., Juusola J.L., Enns E.A., Owens D.K., Garber A.M. (2010). Population strategies to decrease sodium intake and the burden of cardiovascular disease: A cost-effectiveness analysis. Ann. Intern. Med..

[74] Webb M., Fahimi S., Singh G.M., Khatibzadeh S., Micha R., Powles J., Mozaffarian D. (2017). Cost effectiveness of a government supported policy strategy to decrease sodium intake: Global analysis across 183 nations. BMJ.

[75] Palmer B.F. (2009). Hyponatremia in the intensive care unit. Semin. Nephrol..

[76] Mancia G., Kreutz R., Brunström M., Burnier M., Grassi G., Januszewicz A., Muiesan M.L., Tsioufis K., Agabiti-Rosei E., Algharably E.A.E. (2023). 2023 ESH Guidelines for the management of arterial hypertension The Task Force for the management of arterial hypertension of the European Society of Hypertension Endorsed by the International Society of Hypertension (ISH) and the European Renal Association (ERA). J. Hypertens..

[77] Bull N.L., Buss D.H. (1990). Contribution of foods to sodium intakes. Proc. Nutr. Soc..

[78] Vici G., Rosi A., Angelino D., Polzonetti V., Scazzina F., Pellegrini N., Martini D. (2023). on behalf of the SINU Young Working Group. Salt content of prepacked cereal-based products and their potential contribution to salt intake of the italian adult population: Results from a simulation study. Nutr. Metab. Cardiovasc. Dis..

[79] Aureli V., Rossi L. (2022). Nutrition Knowledge as a Driver of Adherence to the Mediterranean Diet in Italy. Front. Nutr..

[80] Noah A., Truswell S. (2003). Commodities consumed in Italy, Greece and other Mediterranean countries compared with Australia in 1960s and 1990s. Asia Pac. J. Clin. Nutr..

[81] Garcia-Closas R., Berenguer A., González C.A. (2006). Changes in food supply in Mediterranean countries from 1961 to 2001. Public Health Nutr..

[82] Lopez C.N., Martinez-Gonzalez M.A., Sanchez-Villegas A., Alonso A., Pimenta A.M., Bes-Rastrollo M. (2009). Costs of Mediterranean and western dietary patterns in a Spanish cohort and their relationship with prospective weight change. J. Epidemiol. Community Health.

[83] Aggarwal A., Monsivais P., Cook A.J., Drewnowski A. (2011). Does diet cost mediate the relation between socioeconomic position and diet quality?. Eur. J. Clin. Nutr..

[84] Buckland G., Bach A., Serra-Majem L. (2008). Obesity and the Mediterranean diet: A systematic review of observational and intervention studies. Obes. Rev..

[85] Shrewsbury V., Wardle J. (2008). Socioeconomic status and adiposity in childhood: A systematic review of cross-sectional studies 1990–2005. Obesity.

